# Development of a risk prediction score and equation for chronic kidney disease: a retrospective cohort study

**DOI:** 10.1038/s41598-023-32279-z

**Published:** 2023-03-27

**Authors:** Shin Kawasoe, Takuro Kubozono, Anwar Ahmed Salim, Haruhito Yoshimine, Seiichi Mawatari, Satoko Ojima, Takeko Kawabata, Yoshiyuki Ikeda, Hironori Miyahara, Koichi Tokushige, Akio Ido, Mitsuru Ohishi

**Affiliations:** 1 Department of Cardiovascular Medicine and Hypertension, Graduate School of Medical and Dental Sciences, Kagoshima University, 8-35-1 Sakuragaoka, Kagoshima, 890-0075 Japan; 2 Digestive and Lifestyle Diseases, Department of Human and Environmental Sciences, Kagoshima University Graduate School of Medical and Dental Sciences, Kagoshima, Japan; 3 Kagoshima Kouseiren Hospital, Kagoshima, Japan

**Keywords:** Kidney diseases, Risk factors

## Abstract

Chronic kidney disease (CKD) is a risk factor for end-stage renal disease and contributes to increased risk of cardiovascular disease morbidity and mortality. We aimed to develop a risk prediction score and equation for future CKD using health checkup data. This study included 58,423 Japanese participants aged 30–69 years, who were randomly assigned to derivation and validation cohorts at a ratio of 2:1. The predictors were anthropometric indices, life style, and blood sampling data. In derivation cohort, we performed multivariable logistic regression analysis and obtained the standardized beta coefficient of each factor that was significantly associated with new-onset CKD and assigned scores to each factor. We created a score and an equation to predict CKD after 5 years and applied them to validation cohort to assess their reproducibility. The risk score ranged 0–16, consisting of age, sex, hypertension, dyslipidemia, diabetes, hyperuricemia, and estimated glomerular filtration rate (eGFR), with area under the curve (AUC) of 0.78 for the derivation cohort and 0.79 for the validation cohort. The CKD incidence gradually and constantly increased as the score increased from ≤ 6 to ≥ 14. The equation consisted of the seven indices described above, with AUC of 0.88 for the derivation cohort and 0.89 for the validation cohort. We developed a risk score and equation to predict CKD incidence after 5 years in Japanese population under 70 years of age. These models had reasonably high predictivity, and their reproducibility was confirmed through internal validation.

## Introduction

Progressive kidney dysfunction leads to end-stage kidney disease (ESKD), requiring dialysis or transplantation. The number of patients requiring dialysis in Japan continues to increase, exceeding 330,000^1^. Patients with chronic kidney disease (CKD) have an increased relative risk of coronary heart disease, heart failure, and stroke compared to those without CKD^2–5^. ESKD and cardiovascular diseases secondary to the renal impairment have become important medical problems that lead to a decline in the quality of life and increased national health care costs.

A changing lifestyle and an aging population have dramatically altered each pathogenetic mechanism contributing to renal impairment. According to data from the Japanese population, diabetic nephropathy was the most common primary disease among patients requiring dialysis (39.0%), followed by chronic glomerulonephritis (27.8%), and nephrosclerosis (10.3%)^1^. The incidence of diabetic nephropathy and nephrosclerosis is increasing annually, while the incidence of chronic glomerulonephritis is declining^1^. Lifestyle and the coexistence of other lifestyle-related diseases play a major role in renal dysfunction. It is very important to focus on risk factors to identify patients who may develop renal dysfunction in the future, because improving renal function is difficult after deterioration. Prophylactic intervention in a high-risk population with renal dysfunction may reduce progression and ESKD and cardiovascular mortality^6^. In developed countries, CKD is generally associated with old age, diabetes, hypertension, obesity, and cardiovascular disease^7^. Some of these can be improved by promoting lifestyle and behavioral changes. It has been reported that smoking cessation inhibited the progression of CKD. In diabetic patients with normal renal function and albuminuria, smoking cessation has been reported to decrease albuminuria^8^. Metabolic syndrome control were independently associated with a lesser progression of diabetic nephropathy^9^. In Japan, since 2008, people aged ≥ 40 years are recommended to undergo regular health checkups to prevent lifestyle-related diseases. It would be clinically useful to find a way to predict CKD using variables measured during these checkups.

Several studies have developed predictive scores for CKD, but they differ in the target population’s ethnicity and score complexity, determined by the number and classification of factors^10,11^. Since the prevalence of renal dysfunction and mechanisms associated with its development vary by race, a unique Japanese scoring system is needed to predict CKD in the Japanese population. Although Nelson’s model included four cohorts of Japanese, they represented only 1.6% of the total participants^11^. Furthermore, although a complex scoring system may provide accurate prediction, it is inconvenient in daily clinical practice. Therefore, we created a simple risk score and an equation for incident CKD after 5 years using Japanese large-scale health checkup data, and evaluated its internal validity.

## Methods

### Data and study population

This study was conducted in the same population as the participants for whom we previously created a risk model for developing hypertension^12^. Data were collected from participants aged 30–69 years after annual health checkups at Kagoshima Kouseiren Hospital between April 2008 and March 2016. We selected participants whose data were available at baseline and after 5 years (range, 3–7 years). Participants were excluded if they had CKD, defined as an estimated glomerular filtration rate (eGFR) < 60 mL/min/1.73 m^2^; were undergoing dialysis therapy; were post-renal transplantation at baseline; and those with missing data.

Two-thirds of the participants were randomly assigned to the derivation cohort to generate a score and an equation to predict CKD. The remaining one-third was used as a validation cohort to assess the validity of the score and equation obtained in the derivation cohort.

This study was conducted in accordance with the Declaration of Helsinki and was approved by the Institutional Ethics Committees of the Graduate School of Medical and Dental Sciences, Kagoshima University. The Ethics Committee has approved that since only existing anonymized data were used in this study, it is not necessary to obtain the informed consent of each individual.

### Risk factors

Age was categorized into four groups: 30–39, 40–49, 50–59, and 60–69 years. Height and weight were measured using standard anthropometric methods. Body mass index (BMI) was calculated as weight (kg) divided by height squared (m^2^) and categorized into ≤ 24.9 kg/m^2^ and ≥ 25.0 kg/m^2^ groups.

The following categories were determined by a self-administered questionnaire: smoker (currently smoker) or non-smoker (have never smoked or smoked in the past), non-frequent drinker (drinking less than 10 days per month) or frequent drinker (drinking more than 10 days per month), habit exerciser (30 min or more a day). Data on drugs for hypertension, diabetes, dyslipidemia, and hyperuricemia were collected using a self-administered questionnaire. Blood samples were obtained after an overnight fast. Serum lipids, glucose, uric acid, and creatinine levels were measured using standard laboratory procedures. Underlying diseases were defined as follows: hypertension (under treatment with antihypertensive agents or blood pressure ≥ 140/90 mmHg), diabetes (under treatment with oral hypoglycemic agents or insulin, or fasting blood glucose ≥ 126 mg/dL), dyslipidemia (under treatment with lipid-lowering agents, serum triglycerides ≥ 150 mg/dL, serum low-density lipoprotein-cholesterol ≥ 140 mg/dL, or serum high-density lipoprotein-cholesterol < 40 mg/dL), and hyperuricemia (under treatment with uric acid lowering agents or serum uric acid level > 7.0 mg/dL). The eGFR was determined according to the new Japanese coefficient for the modified isotope dilution mass spectrometry-traceable Modification of Diet in Renal Disease study equation:

$$ {\text{eGFR}} = {194} \times {\text{SCr}}^{{ - {1}.0{94}}} \times {\text{Age}}^{{ - 0.{287}}} . $$

For women, eGFR was multiplied by a correction factor of 0.739^13^. The baseline renal function was categorized into four groups by baseline eGFR: 60.0–69.9, 70.0–79.9, 80.0–89.9, and ≥ 90.0 mL/min/1.73 m^2^.

The outcome was CKD at the 5-year follow-up, defined as eGFR < 60.0 mL/min/1.73 m^2^.

### Statistical analysis

As in our previous risk model for developing hypertension, we used a similar analytical method^12^. Continuous variables (age, BMI, blood pressure, and hematological parameters) are expressed as mean ± standard deviation, except for skewed distributed indices including triglyceride and blood glucose levels, which are expressed as median (1st quartile, 3rd quartile). Categorical variables, including underlying diseases and lifestyle variables, are expressed as proportions (percentages). Differences between the derivation and validation cohorts for normally distributed continuous variables, skewed-distribution continuous variables, and categorical variables were analyzed using the Student’s unpaired t-test, Wilcoxon test, and χ^2^ test, respectively.

Univariable and multivariable logistic regression analyses were performed for each variable to estimate the odds ratio and 95% confidence interval for CKD incidence. To create a risk score that predicts 5-year incidence of CKD, the following scores corresponding to standardized beta coefficients were assigned to each risk factor category for items that were significant in the multivariable logistic regression analysis, based on the methodology used in the Japan Epidemiology Collaboration on Occupational Health Study Group’s study: 1, β = 0.01–0.20; 2, β = 0.21–0.80; 3, β = 0.81–1.20; 4, β = 1.21–2.20; and 5, β > 2.20^14–16^. The reference category for each variable was given a point of 0, and the risk score for developing CKD was calculated as the sum of the individual points.

The discriminative performance of the score was assessed using the area under the curve (AUC) from the receiver operating characteristic (ROC) analysis. The performance of each score was assessed using sensitivity, specificity, positive predictive value, negative predictive value, and the Youden index^16^. The consistency of association between the scores and CKD incidence was evaluated using the Cochran–Armitage trend test. Then, the score was applied to the validation cohort and ROC analysis was performed.

To compensate for the weakness of the study being derived from data from a single institution, a sensitivity analysis was performed using the bootstrap resample method. The 95% bootstrap confidence interval of the odds ratios in multivariable logistic regression analysis were calculated based on 2500 bootstrap resamples. We then used the β coefficients for the risk factors that were significant in the logistic regression analysis to create an equation that directly calculates the proportion of CKD after 5 years, and assessed its predictive ability and reproducibility in the same way as for the score. In developing the equation, continuous variables such as age and eGFR were used as continuous values without categorization. We evaluated the calibration using calibration plots.

All statistical analyses were performed using JMP Pro version 14 (SAS Institute, Cary, NC, USA) for Windows. Statistical significance was set at P < 0.05.

## Results

### Baseline characteristics

Overall, 167,706 participants aged 30–69 years underwent medical examinations at least once during the study period. Among these, 71,002 individuals had available 5-year follow-up data. A total of 12,579 individuals were excluded (baseline CKD, 7,871; missing variables, 4,708). Finally, the data of 58,423 participants (age, 53.8 ± 10.2 years; male, 50.0%) was analyzed.

Table 1 shows the baseline characteristics of the derivation and validation cohorts. The age and proportion of men were 53.8 ± 10.2 years and 49.8% in the derivation cohort and 53.8 ± 10.2 years and 50.1% in the validation cohort, respectively. After the follow-up period (median, 5.0 years; 1st quartile, 4.7 years; 3rd quartile, 5.1 years), we identified 2,679 (6.9%) and 1,319 (6.8%) cases of new-onset CKD in the derivation and validation cohorts, respectively.

**Table 1:** Baseline characteristics of study population in the derivation and validation cohorts.

	Derivation cohort	Validation cohort
	N = 38,948	N = 19,475
Age, years	53.8 ± 10.2	53.8 ± 10.2
Men, %	49.8	50.1
BMI, kg/m2	23.3 ± 3.4	23.3 ± 3.4
SBP, mmHg	123.8 ± 18.4	124.0 ± 18.1
DBP, mmHg	76.6 ± 11.3	76.6 ± 11.2
Hypertension, %	32.5	32.5
Diabetes, %	8.5	8.3
Dyslipidemia, %	45.9	45.4
Hyperuricemia, %	11.2	11.2
Current smoking, %	20.9	21
Frequent drinking, %	13.2	13.1
Creatinine, mg/dL	0.71 ± 0.14	0.71 ± 0.14
eGFR	79.8 ± 12.4	79.7 ± 12.5
Uric acid, mg/dL	5.1 ± 1.4	5.1 ± 1.4
Triglyceride, mg/dL	89 [63, 129]	89 [64, 130]
LDL-C, mg/dL	122.8 ± 31.2	122.6 ± 31.0
HDL-C, mg/dL	60.4 ± 14.9	60.4 ± 14.8
BG, mg/dL	95 [89, 103]	95 [89, 103]

Baseline characteristics of study population in the derivation and validation cohorts. Continuous variables are expressed as mean ± standard deviation, except for triglyceride and blood glucose levels, which are expressed as median [1st quartile, 3rd quartile]. Categorical variables, including cardiovascular risk factors and lifestyle variables, are expressed as number of subjects and proportions (percentages).

*BMI* body mass index, *SBP* systolic blood pressure, *DBP* diastolic blood pressure, *eGFR* estimated glomerular filtration rate, *LDL-C* low-density lipoprotein cholesterol, *HDL-C* high-density lipoprotein cholesterol, *BG* blood pressure.

### Association between risk factors and incident CKD

The association between incident CKD and risk factor candidates is shown in Table 2. In the univariable model, older age, male sex, higher BMI, non-current smoking, non-frequent alcohol drinking, hypertension, diabetes, dyslipidemia, hyperuricemia, and lower eGFR were associated with an increased risk of CKD. In the multivariable model, older age, male sex, non-frequent drinking, hypertension, diabetes, dyslipidemia, hyperuricemia, and lower eGFR were significantly associated with an increased CKD risk. Our risk models included age, gender, hypertension, diabetes, dyslipidemia, hyperuricemia, and eGFR. Considering the lack of sufficient information on alcohol consumption, it was not included in the risk model components.

**Table 2:** Odds ratios and 95% confidence interval of 5-year incidence of CKD for each risk factor.

Risk factors		No. of subjects	No. of cases (%)	Univariable	Multivariable
				OR (95% CI)	OR (95% CI)
Age, yars	30–39	4532	64 (1.4)	Ref	Ref
	40–49	8405	280 (3.3)	2.41 (1.83–3.16)	1.44 (1.08–1.90)
	50–59	11,805	736 (6.2)	4.64 (3.59–6.01)	2.03 (1.55–2.65)
	60–69	14,206	1599 (11.3)	8.85 (6.88–11.39)	3.09 (2.37–4.03)
Sex	Men	19,403	1433 (7.4)	1.17 (1.08–1.27)	1.19 (1.08–1.32)
	Women	19,545	1246 (6.4)	Ref	Ref
BMI, kg/m2	< 24.9	28,315	1843 (6.5)	Ref	Ref
	> 25.0	10,633	836 (7.9)	1.23 (1.13–1.33)	1.04 (0.95–1.15)
Current smoking	No	30,809	2248 (7.3)	1.41 (1.27–1.56)	Ref
	Yes	8139	431 (5.3)	Ref	1.05 (0.93–1.19)
Frequent drinking	No	33,815	2391 (7.1)	1.28 (1.13–1.45)	1.19 (1.03–1.36)
	Yes	5133	288 (5.6)	Ref	Ref
Hypertension	No	26,291	1371 (5.2)	Ref	Ref
	Yes	12,657	1308 (10.3)	2.09 (1.94–2.27)	1.45 (1.33–1.58)
Diabetes	No	35,614	2358 (6.6)	Ref	Ref
	Yes	3334	321 (9.6)	1.50 (1.33–1.70)	1.30 (1.14–1.49)
Dyslipidemia	No	21,084	1204 (5.7)	Ref	Ref
	Yes	17,864	1475 (8.3)	1.49 (1.37–1.61)	1.12 (1.03–1.22)
Hyperuricemia	No	34,586	2225 (6.4)	Ref	Ref
	Yes	4362	454 (10.4)	1.69 (1.52–1.88)	1.30 (1.15–1.47)
eGFR	> 90.0	7203	28 (0.4)	Ref	Ref
	75.0–89.9	16,298	211 (1.3)	3.36 (2.26–4.99)	2.94 (1.98–4.37)
	60.0–74.9	15,447	2440 (15.8)	48.1 (33.1–69.8)	36.8 (25.3–53.6)

The odds ratios and 95% confidence intervals of 5-year incidence of CKD for each risk factors using logistic regression analysis. In multivariable model, the odds ratios were adjusted for the following variables: age categories, sex, body mass index categories, current smoking, frequent drinking, hypertension, diabetes, dyslipidemia, hyperuricemia, and eGFR categories.

*BMI* body mass index, *eGFR* estimated glomerular filtration ratio, *OR* odds ratio, *CI* confidence interval.

### Risk prediction score for 5-year incidence of CKD

**Figure 1:**
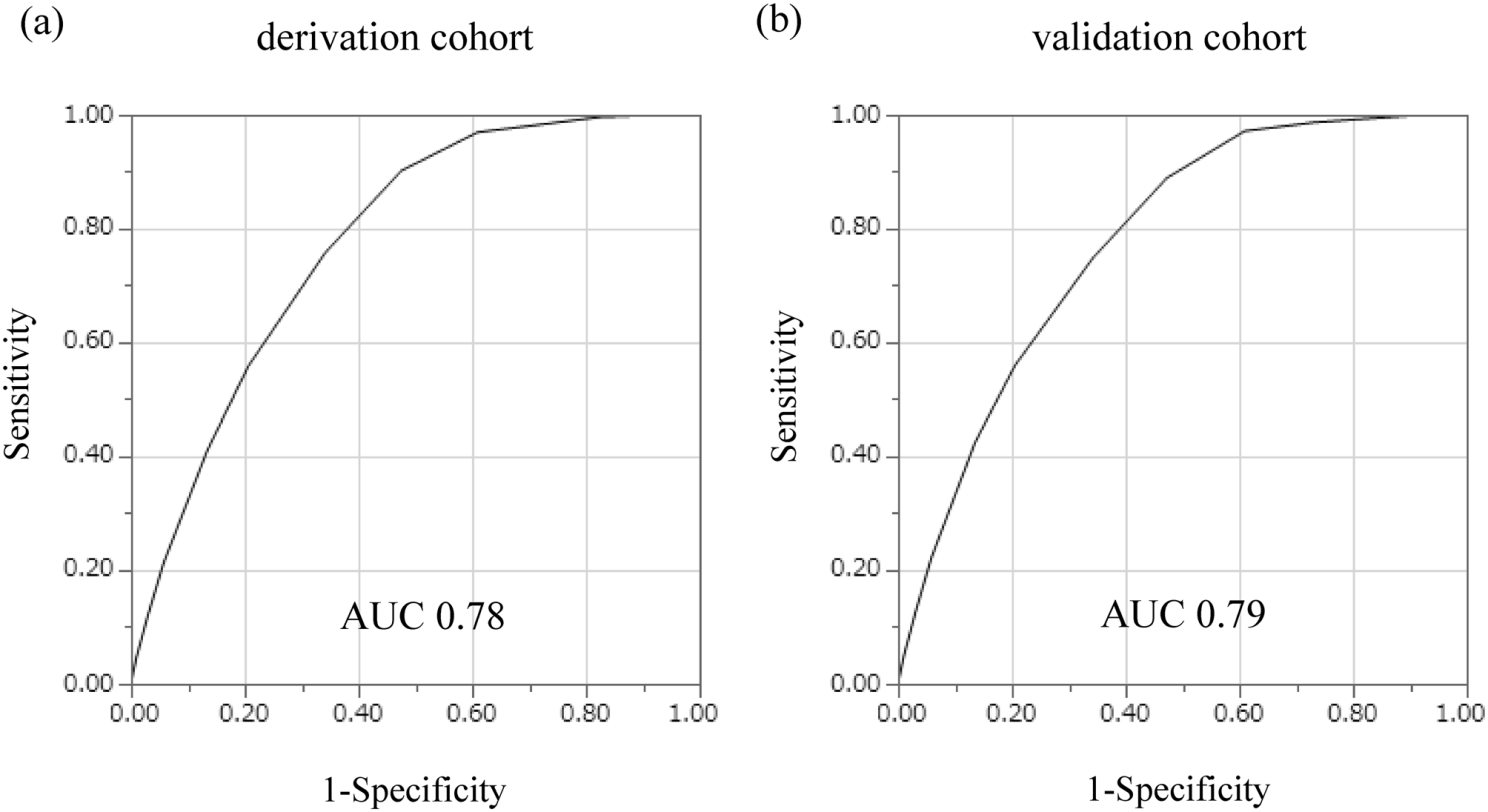
ROC curves in the derivation and validation cohorts for the risk score for predicting the 5-year incidence of CKD. Receiver operating characteristic curves of the derivation cohort (**a**) and validation cohort (**b**) for the risk score to predict the 5-year incidence of CKD. *AUC* area under the curve, *CKD* chronic kidney disease, *ROC* receiver operating characteristic.

**Figure 2:**
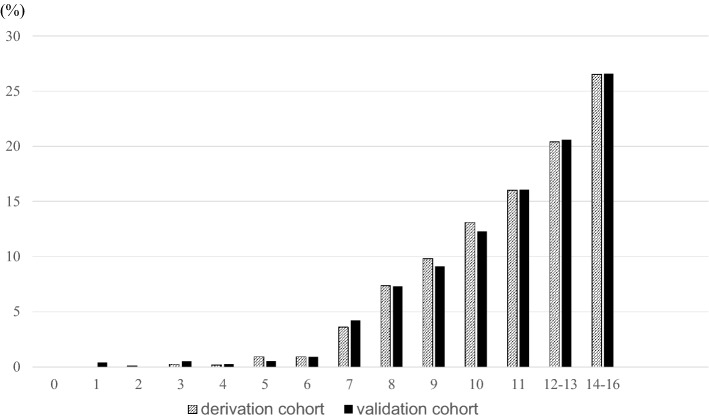
Proportions of developing CKD after 5 years at each score in the derivation and validation cohort. Proportion of CKD development after 5 years for each score in the derivation and validation cohort. The lined bars represent the proportion of 5-year incidence of CKD in the derivation cohort, and the black-shaded bars represent the proportion of 5-year incidence of CKD in the validation cohort. *CKD* chronic kidney disease.

The risk factors and points derived for each category are shown in Table 3. The risk score, sum of all points, could range from 0 to 16. From the ROC curve predicting CKD incidence, the AUC was 0.78 (Fig. 1a). Table 4 shows the predictive performance for a range of cutoff points. A score of ≥ 8 showed the highest Youden index in the derivation cohort, with a sensitivity of 0.90 and specificity of 0.52. The observed incidence of CKD for each score is shown in Fig. 2. At a score of 0 to 6, the observed risk of 5-year CKD was less than 1%. As the score increased, the risk gradually increased (P < 0.001). At a score of 14 to 16, more than 25% of the participants had developed CKD after 5 years.

**Table 3:** Points assigned to predict 5-year incidence of CKD.

Risk factors		β	Point
Age, years	30–39		0
	40–49	0.36	2
	50–59	0.71	2
	60–69	1.13	3
Sex	Women		0
	Men	0.18	1
Hypertension	No		0
	Yes	0.37	2
Diabetes	No		0
	Yes	0.27	2
Dyslipidemia	No		0
	Yes	0.11	1
Hyperuricemia	No		0
	Yes	0.26	2
eGFR,	> 90.0		0
	75.0–89.9	1.08	3
	60.0–74.9	3.61	5

We assigned each category of risk factor with one of the following point scores, corresponding to the standardized β coefficients of multivariate logistic regression,: 1, β = 0.01–0.20; 2, β = 0.21–0.80; 3, β = 0.81–1.20; 4, β = 1.21–2.20; and 5, β > 2.20. The reference category for each variable was given a score of 0. β, standardized regression coefficient.

**Table 4:** Predictive performance of the developed 5-year CKD risk score.

Derivation cohort					
Risk scores	Sensitivity	Specificity	PPV	NPV	Youden index
> 0	1.00	0.00	0.07	1.00	0.00
> 1	1.00	0.02	0.07	1.00	0.02
> 2	1.00	0.03	0.07	1.00	0.03
> 3	1.00	0.07	0.07	1.00	0.07
> 4	1.00	0.13	0.08	1.00	0.12
> 5	1.00	0.17	0.08	1.00	0.17
> 6	0.99	0.27	0.09	0.99	0.25
> 7	0.97	0.39	0.11	0.99	0.36
> 8	0.90	0.52	0.12	0.97	0.43
> 9	0.76	0.66	0.14	0.96	0.42
> 10	0.56	0.79	0.17	0.95	0.35
> 11	0.41	0.87	0.18	0.94	0.28
> 12	0.21	0.94	0.22	0.94	0.15
> 13	0.12	0.97	0.23	0.93	0.09
> 14	0.05	0.99	0.27	0.93	0.04
> 15	0.01	1.00	0.27	0.93	0.01
> 16	0.01	1.00	0.35	0.93	0.01

Validation cohort					
Risk scores	Sensitivity	Specificity	PPV	NPV	Youden index
> 0	1.00	0.00	0.07	1.00	0.00
> 1	1.00	0.02	0.07	1.00	0.02
> 2	1.00	0.03	0.07	1.00	0.03
> 3	1.00	0.07	0.07	1.00	0.07
> 4	1.00	0.12	0.08	1.00	0.12
> 5	0.99	0.17	0.08	1.00	0.17
> 6	0.99	0.27	0.09	0.99	0.26
> 7	0.97	0.39	0.10	0.98	0.36
> 8	0.89	0.53	0.12	0.97	0.42
> 9	0.75	0.66	0.14	0.96	0.41
> 10	0.56	0.79	0.17	0.95	0.35
> 11	0.42	0.86	0.19	0.94	0.29
> 12	0.22	0.94	0.22	0.94	0.16
> 13	0.13	0.97	0.23	0.93	0.10
> 14	0.05	0.99	0.27	0.93	0.04
> 15	0.01	1.00	0.32	0.93	0.01
> 16	0.01	1.00	0.41	0.93	0.00

Predictive performance of the developed 5-year CKD risk score in derivation and validation.

*PPV* positive predictive value, *NPV* negative predictive value.

When the risk score was applied to the validation cohort, the AUC was 0.79, similar to the derivation cohort (Fig. 1b). At the cutoff point of 8 ≥ points, the score had a sensitivity of 0.89 and specificity of 0.53, which was similar to the derivation cohort (Table 4). As the score increased, the risk gradually increased (P < 0.001, Fig. 2).

The results of the sensitivity analysis using the bootstrap method are shown in Table 5. The odds ratios and 95% confidence intervals from the bootstrap method were similar to those from the derivation cohort in the main analysis.

**Table 5:** Mean odds ratios and 95% confidence intervals of multivariable logistic regressin analysis from bootstrap resample method.

Risk factors		OR (95% CI)
Age, years	30–39	Ref
	40–49	1.49 (1.20–1.91)
	50–59	2.08 (1.68–2.63)
	60–69	3.16 (2.57–4.04)
Sex	Men	1.19 (1.08–1.32)
	Women	Ref
BMI, kg/m2	< 24.9	Ref
	> 25.0	1.03 (0.96–1.12)
Current smoking	No	Ref
	Yes	1.02 (0.93–1.12)
Frequent drinking	No	1.18 (1.04–1.31)
	Yes	Ref
Hypertension	No	Ref
	Yes	1.38 (1.28–1.48)
Diabetes	No	Ref
	Yes	1.38 (1.23–1.53)
Dyslipidemia	No	Ref
	Yes	1.11 (1.03–1.20)
Hyperuricemia	No	Ref
	Yes	1.32 (1.20–1.46)
eGFR	> 90.0	Ref
	75.0–89.9	2.76 (2.06–3.90)
	60.0–74.9	37.6 (28.5–52.3)

The odds ratios and 95% confidence intervals of 5-year incidence of CKD for each risk factors using logistic regression analysis with 2500 bootstrap resampling. In multivariable model, the odds ratios were adjusted for the following variables: age categories, sex, body mass index categories, current smoking, frequent drinking, hypertension, diabetes, dyslipidemia, hyperuricemia, and eGFR categories.

*BMI* body mass index, *OR* odds ratio, *CI* confidence interval.

### Risk prediction equation for 5-year incidence of CKD

We developed an equation to predict CKD probability after 5 years, using age (years old), sex (female, 0; male, 1), hypertension (no, 0; yes, 1), dyslipidemia (no, 0; yes, 1), diabetes (no, 0; yes, 1), hyperuricemia (no, 0; yes, 1), and eGFR (mL/min/1.73 m^2^).

#### Probability of 5-year incidence of CKD

Probability = 1/ (1 + exp[−{9.4876 + 0.0311 × age + 0.2400 × sex + 0.3470 × hypertension + 0.0893 × dyslipidemia + 0.3444 × diabetes + 0.0832 × hyperuricemia + -0.1980 × eGFR}]).

**Figure 3:**
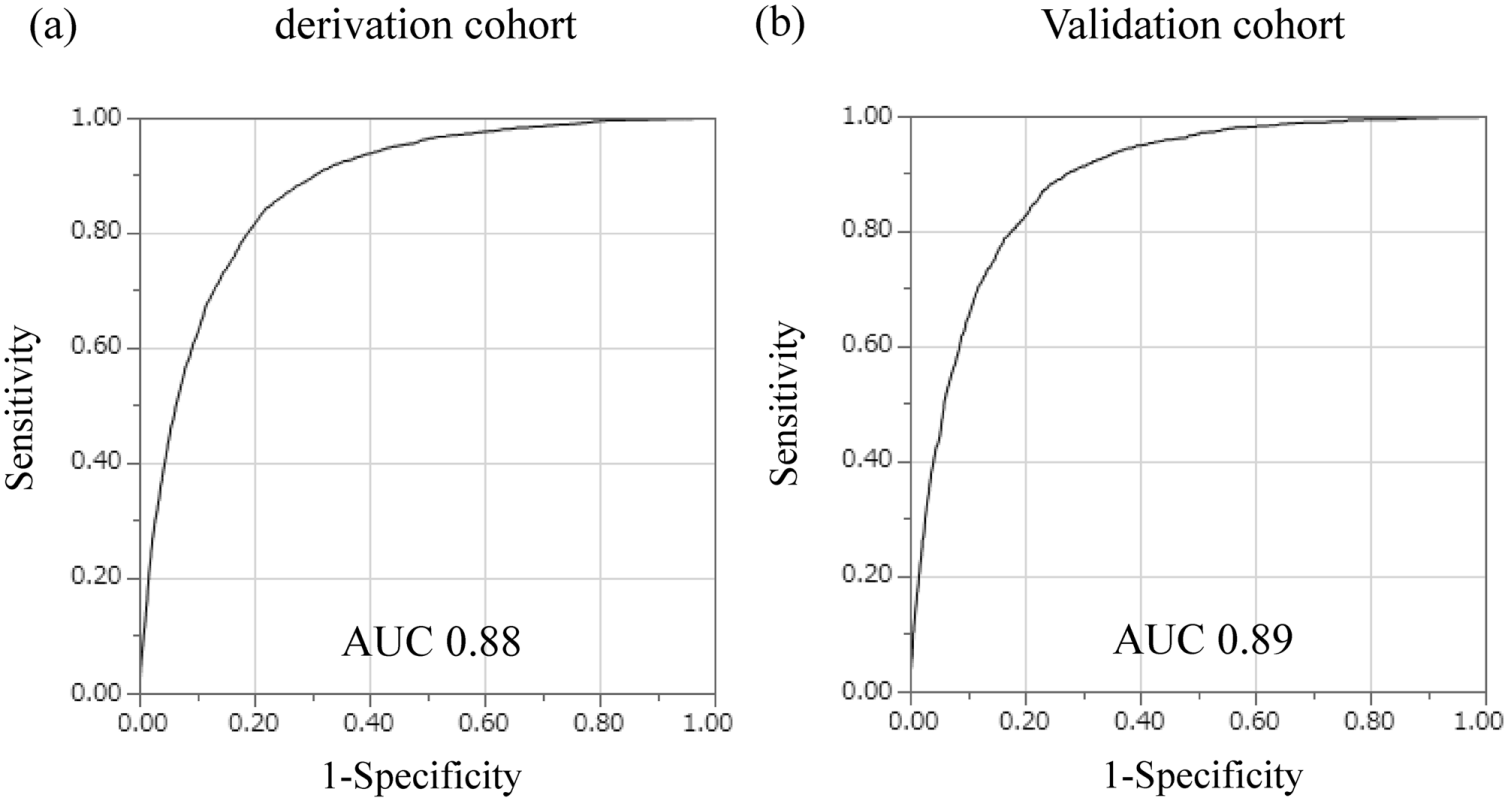
ROC curves in the derivation and validation cohorts for the risk equation for predicting the 5-year incidence of CKD. Receiver operating characteristic curves of the derivation cohort (**a**) and validation cohort (**b**) for the risk equation to predict the 5-year incidence of CKD. *AUC* area under the curve, *CKD* chronic kidney disease, *ROC* receiver operating characteristic.

**Figure 4:**
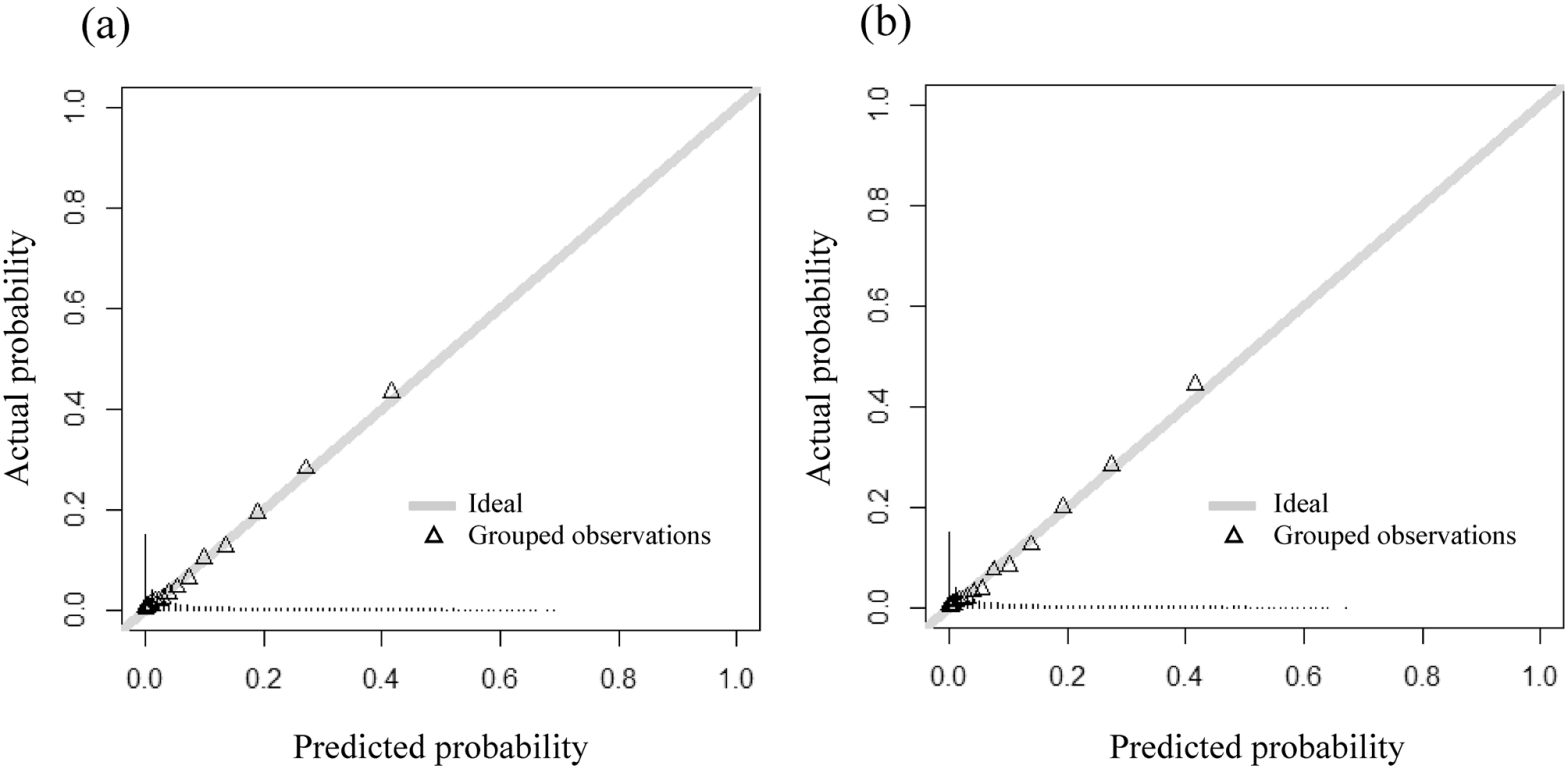
Calibration plots for the equation model in derivation and validation cohorts. The visual agreement between the CKD predictions (Predicted probability) and observations (Actual probability) for the equation model in the derivation and validation cohorts. (**a**) derivation cohort (**b**) validation cohort.

The median probability obtained from the derivation cohort was 0.018 (interquartile range 0.002–0.084), and the AUC value of the ROC curve for the development of CKD after 5 years was 0.88, with a sensitivity of 0.84 and a specificity of 0.78 at a cutoff value of 0.077 calculated from the Youden index (Fig. 3a). When the risk equation was applied to the validation cohort, the AUC was 0.89, with a sensitivity of 0.87 and a specificity of 0.77 with a cutoff value of 0.075 (Fig. 3b). The results of the calibration are shown in Fig. 4. Good visual calibration is achieved for both the derivation and validation cohorts.

## Discussion

We developed a score and an equation and to predict the 5-year risk of incident CKD defined as eGFR < 60 mL/min/1.73 m^2^ based on seven indicators (age, sex, hypertension, diabetes, dyslipidemia, hyperuricemia, and eGFR) from large-scale health checkup data. CKD incidence increased with increasing risk scores, and the same predictive ability was validated when applied to population not included in the score development. We have achieved our goal of creating risk models based on simple indexes that is applicable to the Japanese population under 70 years of age and can be easily used in clinical practice.

Several studies have reported risk scores for predicting future renal dysfunction. O'Seaghdha et al. studied the Framingham Offspring Study cohort of Caucasians and developed a score (0–15 points) to predict CKD incidence (eGFR < 60 mL/min/1.73 m^2^) at 10 years based on age, hypertension, diabetes, baseline eGFR category, and the presence of proteinuria using a test paper method. The c-statistic for the CKD risk score was 0.74 when validated using an external cohort of whites and blacks, and the predictive ability was good even when for only blacks^10^. Predictive equations for CKD using data from over 5 million people in 34 cohort studies from 28 countries have also been reported. In addition to age and sex, race/ethnicity, eGFR, history of cardiovascular disease and hypertension, smoking history, BMI, and urinary albumin are used for calculation. For diabetic patients, glycated hemoglobin and the use of diabetic medications were added. The C statistic was 0.845 in the absence of diabetes and 0.801 in the presence of diabetes^11^. We should apply these prior risk models to our study population and compare the predictive abilities. However, these two risk models could not be applied to our study population because they require urinary protein and albumin information. The AUC and c-statistic of the ROC curve are commonly used as indicators of the discriminative ability of risk scores. The model developed in this study had an AUC of 0.79 for the score and 0.89 for the equation. Although direct comparison with previous reports was not possible, the discriminative ability of our model is somewhat superior.

As described above, several risk prediction models have been reported to predict the development of CKD in various ethnic groups in different countries. Since CKD prevalence and its risk factors varies by race, it is preferable to use the prediction model determined from Japanese data to predict CKD risk in Japanese people. Although Nelson et al. included four cohorts of Japanese, they represented only 1.6% of the total participants and included data as old as the 1970s, whereas the present study included only Japanese and used relatively recent data from 2008 to 2016 to develop the score and equation. These factors may have contributed to the improvement in discrimination. In Japan, all men and women aged ≥ 40 years are recommended to undergo annual specific health checkups and receive guidance focusing on visceral obesity if needed. We aimed to create a prediction model based on anthropometric measurements, blood tests, questionnaires, and other data obtained from these checkups. We achieved a good prediction accuracy of AUC 0.78 for the risk score and 0.88 for the risk equation. The risk score obtained in this study can be easily calculated by healthcare workers in daily clinical practice, enabling patients to understand the extent of their risk and contribute to decision-making for treatment and lifestyle improvement. The risk prediction equation has the advantage of more accurately predicting CKD risk, but they are not easy to calculate and should be considered for practical application in the form of online calculators or others. In addition, development of an application that can calculate this risk score would be more convenient and clinically useful for assessing risk.

Age-associated loss of kidney function has been recognized for decades. With aging, many participants exhibit a progressive decrease in the glomerular filtration rate and renal blood flow. The decrease in glomerular filtration rate is due to a reduction in the glomerular capillary plasma flow rate and glomerular capillary ultrafiltration coefficient^17^. The proportion of participants over 70 years who had a medical checkup after 5 years was low. In addition, 40% of the participants had already developed CKD, making the number of cases available for analysis small. Therefore, participants over 70 years were excluded from this study. Previous epidemiologic studies indicate that the incidence of ESKD is higher in men than in women^18^. A recent study found that female mice were more tolerant to ischemic-reperfusion injury than male mice and that female mice receiving supplemental estrogen before ischemia were further protected^19^. The possible mechanisms underlying the reno-protective role in females seem to be related to estrogen. This is supported by clinical studies demonstrating that premenopausal women are better protected from renal and cardiovascular disease compared to age-matched men; this protective effect seems to be lost with aging and menopause^20^. However, the mechanisms by which estrogen confers protective renal effects are not well understood.

Hypertension has been reported to be a risk factor for both CKD and ESKD^21,22^. Systemic hypertension causes an increase in intraglomerular capillary pressure, leading to glomerulosclerosis and loss of renal function^22^. CKD is associated with specific qualitative and quantitative lipid abnormalities, resulting in specific dyslipidemias^23–25^. Specific abnormalities in lipoprotein metabolism, caused by the inappropriate activity of some key enzymes and metabolic pathways, develop in the early stages of renal failure and result in dyslipidemia, which is a risk factor for the development of atherosclerosis. As CKD progresses, dyslipidemia worsens and may adversely affect renal function through the promotion of atherosclerosis^23,26,27^. Iseki et al. found a significant association between elevated serum uric acid and serum creatinine levels in a Japanese cohort study^28^. Further, high uric acid level (≥ 6.0 mg/dL) was a risk factor for ESKD development in women^29^. It is suggested that hyperuricemia may damage vascular endothelial cells and contribute to renal dysfunction through the production of reactive oxygen species by xanthin oxide reductase, activation of the renin-angiotensin system, and induction of inflammation by inflammasome activation^30,31^. Although alcohol consumption was a significant factor in the multivariate analysis, we did not include it as a risk factor in this study because we do not have sufficient information on it. In the questionnaires, the frequency per month and daily intake regarding alcohol consumption were asked as separate items. Therefore, we were unable to find an appropriate way to integrate frequency and quantity of intake into a single variable, "alcohol intake“. The association between alcohol intake and CKD is not simple. Moderate alcohol intake (20–40 g ethanol/day) is not a risk factor for CKD^32^, but rather inhibits its progression^33^. On the other hand, heavy alcohol intake (> 60 g/day of ethanol) has been reported to be a risk factor for CKD^34^. For these reasons, we decided that it would be difficult to use the data with alcohol intake not clearly defined.

In addition to the above, this study had several limitations. First, the data were not collected prospectively, and the results should be further validated in a prospective observational study. Second, the participants were limited to those who underwent health examinations at a single institution in Japan. Furthermore, participants tend to be highly interested in their own health, so selection bias cannot be avoided. Third, since we do not have information on the etiology of nephropathy, we do not know if these models work better in predicting nephrosclerosis or diabetic nephropathy compared to glomerulonephritis. Fourth, although proteinuria is itself an important risk factor for the development of CKD, urinary protein was not addressed in this study because of the low rate of obtaining urinalysis results in our participants' data. Finally, due to the large population that was randomly divided into derivation and the validation cohort, both cohorts had nearly identical characteristics. The internal validation therefore has limited informative value and external validation is necessary to assess the discriminative and predictive abilities of scores in real life.

In conclusion, we have developed a clinical risk score and equation to predict the occurrence of CKD after 5 years in a Japanese population under 70 years of age, using age, sex, hypertension, diabetes, dyslipidemia, hyperuricemia, and eGFR levels. Predictive ability was comparable with previous reports, and reproducibility was confirmed through internal validation. These prediction methods could be simple and useful tools for detecting and recognizing high-risk CKD patients in Japan and for providing appropriate counseling.

## Data Availability

The Kagoshima University Institutional Review Board and Kagoshima Kouseirin Hospital applies the restriction for public data sharing due to ethical and legal restrictions of the annual health check-up data containing sensitive information and that participant did not consent to public sharing. The deidentified data may be partly available upon ethical approval by request directed to Dr. Takuro Kubozono (kubozono@m.kufm.kagoshima-u.ac.jp).
